# Cancer Monitoring Methods

**DOI:** 10.1155/2014/981495

**Published:** 2014-04-03

**Authors:** Aleksandra Nikolic, Oronza Antonietta Botrugno, Jelena Urosevic, Natasa Tosic

**Affiliations:** ^1^Laboratory for Molecular Biology, Institute of Molecular Genetics and Genetic Engineering, University of Belgrade, Vojvode Stepe 444A, 11010 Belgrade, Serbia; ^2^Drug Discovery Unit, Department of Experimental Oncology, European Institute of Oncology, IFOM-IEO Campus, Via Adamello 16, 20139 Milan, Italy; ^3^Growth Control and Cancer Metastasis Laboratory, Institute for Research in Biomedicine (IRB Barcelona), Parc Cientific de Barcelona, Carrer de Baldiri Reixac 10, 08028 Barcelona, Spain; ^4^Laboratory for Molecular Hematology, Institute of Molecular Genetics and Genetic Engineering, University of Belgrade, Vojvode Stepe 444A, 11010 Belgrade, Serbia

Methods for cancer monitoring and early detection of the disease are of the utmost importance and represent one of the most active areas of current research. Cancer monitoring is crucial not only for early initial diagnosis of the disease, but also for followup of therapy outcome. Despite being well developed, most methods for cancer monitoring are unsuitable for clinical use because they either are insufficiently accurate, not sensitive enough, or require a lengthy complicated analysis. There is a great necessity for more effective cancer monitoring methods that can improve cancer management in routine clinical setting and increase treatment effectiveness. Advances in this field of research are based on a more detailed understanding of the fundamental biological mechanisms involved in the disease process, as well as on advances in genomic, transcriptomic, proteomic, and metabolomic research.

This special issue encompasses articles on the state of the art, advantages and disadvantages, current limitations, and future perspectives of cancer monitoring methods. Dr. D. Musio with colleagues and Dr. B. Kasper with colleagues present advanced imaging methods in clinical followup of response to therapy. Dr. G. Bertino with colleagues, as well as Dr. P. Mirabelli and Dr. M. Incoronato, offer new insight into the use of some traditional cancer biomarkers in clinical and laboratory practice. The works of Dr. P. Sadlecki with colleagues, Dr. R. Zappacosta with colleagues, and Dr. S. Farivar with colleagues deal with the analysis of protein cancer biomarkers in different types of samples. Of special interest are the articles that report on development, implementation, and validation of novel techniques for cancer monitoring, by Dr. C.-F. Chen with colleagues and Dr. I. Macchia and colleagues.

We would like to thank the authors for their excellent contributions to this special issue. We hope that this issue will be useful to the experts of all profiles dealing with cancer in both clinical and laboratory setting.

*Aleksandra Nikolic* *Oronza Antonietta Botrugno* *Jelena Urosevic* *Natasa Tosic*

